# Association of Tooth Scaling with Acute Myocardial Infarction and Analysis of the Corresponding Medical Expenditure: A Nationwide Population-Based Study

**DOI:** 10.3390/ijerph18147613

**Published:** 2021-07-17

**Authors:** Yi-Wei Kao, Ben-Chang Shia, Huei-Chen Chiang, Mingchih Chen, Szu-Yuan Wu

**Affiliations:** 1 Graduate Institute of Business Administration, College of Management, Fu Jen Catholic University, New Taipei City 242, Taiwan; kyw498762030@gmail.com (Y.-W.K.); 025674@mail.fju.edu.tw (B.-C.S.); huizhen0128@gmail.com (H.-C.C.); 2 AI Development Centers, Fu Jen Catholic University, New Taipei City 242, Taiwan; 3 Department of Food Nutrition and Health Biotechnology, College of Medical and Health Science, Asia University, Taichung 413, Taiwan; 4 Big Data Center, Lo-Hsu Medical Foundation, Lotung Poh-Ai Hospital, Yilan 265, Taiwan; 5 Division of Radiation Oncology, Lo-Hsu Medical Foundation, Lotung Poh-Ai Hospital, Yilan 265, Taiwan; 6 Department of Healthcare Administration, College of Medical and Health Science, Asia University, Taichung 413, Taiwan; 7 Cancer Center, Lo-Hsu Medical Foundation, Lotung Poh-Ai Hospital, Yilan 265, Taiwan; 8 Centers for Regional Anesthesia and Pain Medicine, Wan Fang Hospital, Taipei Medical University, Taipei 110, Taiwan

**Keywords:** tooth scaling, acute myocardial infarction, medical expenditure

## Abstract

Accumulating evidence has shown a significant correlation between periodontal diseases and systemic diseases. In this study, we investigated the association between the frequency of tooth scaling and acute myocardial infarction (AMI). Here, a group of 7164 participants who underwent tooth scaling was compared with another group of 7164 participants without tooth scaling through propensity score matching to assess AMI risk by Cox’s proportional hazard regression. The results show that the hazard ratio of AMI from the tooth scaling group was 0.543 (0.441, 0.670) and the average expenses of AMI in the follow up period was USD 265.76, while the average expenses of AMI in follow up period for control group was USD 292.47. The tooth scaling group was further divided into two subgroups, namely A and B, to check the influence of tooth scaling frequency on AMI risk. We observed that (1) the incidence rate of AMI in the group without any tooth scaling was 3.5%, which is significantly higher than the incidence of 1.9% in the group with tooth scaling; (2) the tooth scaling group had lower total medical expenditures than those of the other group because of the high medical expenditure associated with AMI; and (3) participants who underwent tooth scaling had a lower AMI risk than those who never underwent tooth scaling had. Therefore, the results of this study demonstrate the importance of preventive medicine.

## 1. Introduction

Dental health affects not only the overall health but also the quality of life of individuals. Accumulating evidence has shown that there is a significant relationship between periodontal diseases and systemic diseases [1]. Poor oral hygiene increases the risk of various diseases, such as cardiovascular disease, coronary heart disease, stroke, hypertension, lung disease, diabetes mellitus, and some cancers. Several studies have reported that cavities, periodontal diseases, and oral cancer add to the inflammatory burden of the human body. According to the World Health Organization, noninfectious chronic diseases could soon become the main cause of disability and death among populations in rapidly aging countries. The most common oral health conditions are missing teeth, dental decay, periodontal disease, xerostomia, and oral cancer. Thus, all countries should implement public policies to eliminate the factors that negatively affect oral health [2].

Studies have reported that poor oral hygiene increases cardiovascular disease risk; however, the association between preventive oral care and reduction in cardiovascular risk has not been discussed. Chen et al. studied the association between tooth scaling and cardiovascular event risks [3]. The findings of their multivariate analysis based on a cohort dataset in the NHIRD support tooth scaling as a means of improving oral hygiene and, consequently, reducing the risks of AMI, stroke, and total cardiovascular events. Studies that have investigated whether the improvement of oral hygiene through tooth scaling could reduce infective endocarditis (IE) risk have reported that the percentage of tooth scaling was lower among individuals with IE than in those without IE. Furthermore, when patients were divided into three groups based on tooth scaling frequency, IE risk was lower in patients who underwent tooth scaling more frequently [4]. Periodontal disease is a serious issue in Taiwan; the prevalence of periodontitis has significantly increased in Taiwan over the past years [5]. There are multiple comorbidities, such as pulmonary, endocrinal, metabolic, cardiovascular, neurological, hematological, and skeletal disorders, associated with periodontal disease [6,7,8]. For example, the increased risk of periodontitis in patients with diabetes is estimated to be 2–3 fold; that is, it increases the risk for periodontitis 2–3 times [9]. Diabetes increases the prevalence of periodontitis, the extent of periodontitis (that is, the number of affected teeth), and the severity of the disease [9]. The diabetes incidence among people aged 20–79 years in Taiwan increased by 15% from 2005 (7.86 per 1000 persons) to 2012 (9.04 per 1000 persons) [10]. Moreover, patients with periodontal treatment exhibited a significantly lower risk of pneumonia than the general population [11]. Hence, there is a need to prevent or implement early treatment of periodontal disease in Taiwan owing to its association with acute or chronic comorbidities.

In Taiwan, the NHI system provides each beneficiary with one dental scaling per six months [12]. Its large-scale database provides good evidence with which to evaluate the effectiveness of dental scaling [12]. Therefore, we aimed to use the average expenses of tooth scaling and AMI treatment, which were both calculated in this study, to encourage Taiwanese citizens to have tooth scaling regularly in order to minimize the potential risk of suffering AMI and the derivative expenses. The research hypothesis of this study is that the future expenses of AMI for Taiwanese citizens who have the tooth scaling will be lower than that of the citizens who do not have the tooth scaling treatment at all. Furthermore, the hazard ratio of those citizens who have the tooth scaling treatment regularly will be lower than other citizens who do not. We analyzed the association between the number of tooth scaling services availed and AMI. We observed that individuals who underwent regular tooth scaling had a lower AMI risk and, thus, lower medical expenditures.

## 2. Materials and Methods

In this study, we analyzed data sourced from the Longitudinal Health Insurance Database (LHID) provided by the Taiwan National Health Research Institute. The LHID includes all original claims data for 1,000,000 beneficiaries, randomly sampled from the registry for beneficiaries of the NHI program for the year 2005. This was a retrospective cohort study that used data from 2000 to 2013 from Taiwan NHIRD. This dataset maintains all records of patients in the outpatient and inpatient departments, medication-related information, and other monetary data, such as payments and premiums. The ID numbers of the payments are encrypted and patient privacy is safeguarded. The ninth edition of the International Statistical Classification of Diseases (ICD-9) was adopted in this study to mark the diseases of the patients. The predefined primary endpoint was the incidence of AMI (ICD9: 410.xx).

The participants were divided into two groups. The first group consisted of participants aged 50–64 years who had undergone full-mouth tooth scaling or localized tooth scaling in 2000. The comparison group comprised those who did not undergo tooth scaling in 2000 or later. Hence, there were no crossover participants in the study design. In addition, participants in the comparison group were matched to those in the first group using a propensity score. Propensity score matching enables the comparison of individuals who are equal in terms of all observed variables and differ only with regard to the treatment condition [13]. The factors selected in this research were age, gender, hypertension (ICD9: 401.xx-405.xx), diabetes mellitus (ICD9: 250.xx), hyperlipidemia (ICD9: 272.xx), coronary artery disease (ICD9: 411.xx-414.xx), and chronic renal disease (ICD9: 580.xx-587.xx). The starting date of this study was the date that the participants underwent tooth scaling. The follow up date for the AMI incidents ended on 12 December 2013.

The tooth scaling group was divided into 2 groups based on the average tooth scaling frequency in the follow up period. Group A represented those participants who underwent tooth scaling less than 1 time per year on average. Group B represented those participants who underwent the treatment 1 or 2 times per year on average. We have removed the people that had >2 free dental scaling treatments per year because the free coverage of tooth scaling provided only 2 dental scaling treatments per year. Therefore, to include the people that have >2 free dental scaling treatments per year would have been unreasonable, and the most likely reason for their inclusion in the LHID is an administrative error in health insurance declaration. Therefore, we excluded the people that have >2 free dental scaling treatments per year because they do not fit the general population.

SAS (version 9.3; SAS Institute, Cary, NC, USA) and R (version 3.6.1; R Foundation for Statistical Computing, Vienna, Austria) were used for statistical analyses. Continuous variables are expressed as mean ± SD. Comparisons among the 2 groups were conducted using an independent t-test for continuous variables and a chi-square test for categorical variables. Data are presented as means and standard deviations or medians and percentages. Cox’s proportional hazards regression analysis was adopted to estimate AMI risk after full-mouth tooth scaling or localized tooth scaling. The study was conducted according to the guidelines of the Declaration of Helsinki and approved by the Institutional Review Board of Taipei Medical University (TMU-JIRB No. 20151033).

## 3. Results

In total, 14,328 participants were enrolled in this study, including 7164 who underwent tooth scaling at least once during the study period and 7164 who did not have any record of tooth scaling. Propensity score matching was performed with tooth scaling as the dependent variable. As shown in Table 1, there were no significant differences in age, gender, hypertension, diabetes mellitus, hyperlipidemia, coronary heart disease, and chronic kidney disease between the two groups.

Here, we analyzed the association between the records of tooth scaling and AMI incidence. During the follow-up period of 13 years, 1.9% of the 7497 participants who had undergone tooth scaling experienced AMI. The incidence of AMI in participants who had never undergone tooth scaling was 3.5% (Table 2). Cox’s proportional hazard regression model showed that AMI risk was significantly lower in the tooth scaling group than in the non-tooth scaling group (HR: 0.543; 95% CI (0.441, 0.670)).

Furthermore, the factor of medical expenditure of the NHI program was added for an advanced analysis. It is known that the expenditure to cure AMI in Taiwan exerts a considerable economic burden on the NHI program. When tooth scaling expenses were counted, the lumpsum expenditure of tooth scaling and AMI treatments (TWD 45,434,662) altogether for the group with tooth scaling records was still lower than that of the comparison group of participants without tooth scaling records (Table 2). It can be discovered from Table 2 that the AMI expenses from the tooth scaling group represented around 1.25% of all expenditure, which is lower than that of 1.55% from the control group. Therefore, an increased frequency of tooth scaling was associated with a lower rate of AMI incidents and a lower cost incurred for the NHI program.

We also assessed the influence of the frequency of tooth scaling on cardiovascular risk. We divided participants aged 50–65 years who had undergone tooth scaling in 2000 into two groups, based on the average number of tooth scaling services availed per year from 2008 to 2013. According to the analysis report (Table 3), there are statistically significant differences in age, and hypertension. The result shows that age is lower in Group B and hypertension is higher in Group A.

Table 4 shows the hazard ratio of AMI between Groups A and B (consisting of participants who underwent tooth scaling in 2000 but had different frequencies of tooth scaling in the follow-up period) and those who had not undergone tooth scaling in 2000 or at any time later. Participants from Groups A and B had a lower AMI risk than those without tooth scaling history did. 

**Figure 1:**
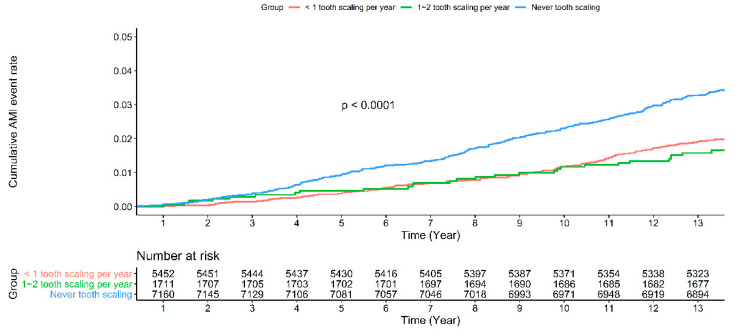
Cumulative acute myocardial infarction rate at 13--year follow up of patients with tooth scaling subgroup or without tooth scaling in the whole cohort.

The hazard risk is shown in Figure 1. Participants from Group B had the lowest AMI risk. The data implied that participants who underwent tooth scaling had lower AMI risk compared with those who never underwent tooth scaling.

We observed that (1) the incidence rate of AMI in the group without any tooth scaling was 3.5%, which is significantly higher than the incidence of 1.9% in the group with tooth scaling; (2) the tooth scaling group had lower total medical expenditures than those of the other group because of the high medical expenditure associated with AMI; and (3) participants who underwent tooth scaling had a lower AMI risk than those who never underwent tooth scaling did.

## 4. Discussion

Although tooth scaling is free of charge in Taiwan, not many individuals avail the service. The National Health Insurance (NHI) of Taiwan has been in operation since March 1995, in which Taiwanese citizens aged ≥12 years are eligible for a complimentary dental checkup and scaling twice a year. However, <50% of Taiwanese residents avail this facility. The Health Promotion Administration and Ministry of Health and Welfare in Taiwan conducted a dental survey involving Taiwanese residents aged ≥12 years, and the results revealed that only 16% of the respondents had undergone tooth scaling in the past 6 months [14]. Another survey conducted in 2013 with 158 respondents aged 65–85 years revealed that only 19% of them underwent regular tooth scaling (once every 6–12 months). Therefore, in the current study, we analyzed the frequency of tooth scaling in Taiwan as well as its association with AMI. Dental scaling in Taiwan is defined as a conventional periodontal therapy, a non-surgical periodontal therapy or deep cleaning, a procedure involving the removal of dental plaque and calculus and then smoothing, or planing, of the surfaces of the roots, removing cementum or dentine that is impregnated with calculus, toxins, or microorganisms, the etiologic agents that cause inflammation [15]. The purpose of this research was to promote the idea that “prevention is better than cure” to strengthen oral healthcare and to reduce periodontal disease risk, subsequently reducing other associated diseases and the corresponding medical expenses.

Several infectious diseases have been implicated in the etiology of myocardial infarction [16]. Similarly, the association between periodontal diseases and heart diseases has often been discussed and several explanations for what underlies this association have been proposed [17,18]. Periodontal disease is a chronic infection that leads to chronic inflammation [19]. Periodontal infection may cause endothelial dysfunction, inflammation, and atherosclerosis [20]. Patients with cardiac arrest incur a large burden of medical expenses [21]. Suchard et al. addressed the issue of medical expenses in their study and indicated that treatment efforts without appropriately diverting medical resources exerted financial pressure on the healthcare system [22]. In the US, the cost of treating AMI was 86.4 billion US dollars in 2016, which was a huge burden. Therefore, an efficient method to prevent AMI is urgently needed to reduce the overall economic burden [23]. Some studies have focused on the medical expenses after suffering from AMI [24,25]. Few studies have emphasized the importance of preventive measures, such as oral care, to reduce AMI risk and the subsequent inpatient and outpatient treatment expenses [26]. Although Taiwan’s NHI program plays a significant role in reducing this financial burden among its citizens, the program itself faces difficulties in long-term stability [27].

The Taiwan NHI program covers nearly 99.9% of Taiwan residents. Owing to the rising costs and rapidly aging population, the system may run out of funds within 2 years, unless increased premiums and other reforms are introduced [28]. Therefore, reduction in the NHI cost and slowing the speed of its financial crisis are the major concerns of this study. The population of Taiwan has not increased much since 1995, indicating a limited increase in premium income. However, Taiwan′s population is aging rapidly. In 2018, individuals aged ≥65 years accounted for 14% of the total population of Taiwan. By 2026, Taiwan is estimated to become a super-aged society, with at least 20% of the population being aged ≥65 years. With a fast-growing population of senior citizens, the Taiwanese NHI program may not have sufficient money from the premium to cover the entire healthcare expenditure. 

Taiwan NHI Research Database (NHIRD) was used to conduct the present research. A previous study has shown that teeth scaling significantly reduces the incidence of cardiovascular diseases [3]. Another study that focused on the association between tooth scaling and the occurrence of atrial fibrillation and infective endocarditis concluded that tooth scaling may reduce the incidence of atrial fibrillation and endocarditis [29]. Furthermore, Chen et al. used the case–crossover design to study the relationship between tooth scaling and infective endocarditis [30]. Although the results showed no significant correlation, the study did not overturn the arguments put forth in previous research. More recent research indicates that tooth scaling plays a role in intracerebral hemorrhage [31].

The strength of our study is that it is the largest and most long-term follow up cohort study that uses well-designed head-to-head propensity score matching to demonstrate that participants who had undergone tooth scaling had a lower AMI risk than those who never underwent tooth scaling had. We have shown that the hazard ratio of AMI from the tooth scaling group was 0.543 (0.441, 0.670) and the average expenses of AMI in the follow up period was USD 265.76, whereas the average expenses of AMI in follow up period for the control group was USD 292.47. Our outcomes could facilitate better financial saving and help to convert the health policy to preventive medicine, advocating for a public health policy that views prevention as better than cure. Additionally, our findings can give government departments a reference for the direction of future health policy.

This study has several limitations. First, the socioeconomic status of the participants was not analyzed. The income, area of residence, and education level may have influenced the health literacy of the participants, which, in turn, is related to willingness to visit a dentist. However, as >99% of Taiwan residents are enrolled in the NHI program and tooth scaling is covered by insurance, the aforementioned factors may not have affected the frequency of tooth scaling. Second, data about personal habits of the participants such as smoking and physical activity were not included. These personal habits are considered important factors that affect AMI risk. However, the study had a large number of participants, which may represent the population statistic of the general public. The influence of these lifestyle factors can also be reduced by matching the history of diseases such as hypertension, diabetes mellitus, hyperlipidemia, coronary heart disease, and chronic kidney disease.

## 5. Conclusions

The current findings suggest that tooth scaling reduces AMI risk. Participants who underwent tooth scaling more frequently had the lowest AMI risk when the most extreme sample of 333 individuals was excluded. Thus, maintaining oral hygiene through regular tooth scaling could help avoid AMI.

The fast-aging population of Taiwan poses several challenges to the financial stability of the NHI program. Promoting the importance of oral health and encouraging the habit of regular tooth scaling among the public might help to reduce the risk of AMI and related expenditure and economic burden on the NHI administration. As previously mentioned, numerous nations, including USA and Japan, have emphasized the importance of oral health and adopted different measures to control the prevalence of periodontal disease and the risk of coronary heart disease, aneurysms, and other health problems. In Taiwan, the free coverage of tooth scaling provided by the NHI program has built a solid base for oral hygiene. The findings of this study show the benefits of tooth scaling in lowering disease risks and relieving the economic tension of NHI.

## Figures and Tables

**Table 1:** Baseline characteristics of participants.

	Tooth Scaling Group	Comparison Group	*p*-Value
n	7164	7164	
Gender = male (%)	3307 (46.2)	3307 (46.2)	1.000
Age (mean (SD))	56.10 (4.34)	56.09 (4.35)	0.820
Hypertension = Yes (%)	1545 (21.6)	1468 (20.5)	0.119
Diabetes mellitus = Yes (%)	741 (10.3)	734 (10.2)	0.869
Hyperlipidemia = Yes (%)	751 (10.5)	749 (10.5)	0.978
Coronary artery disease = Yes (%)	514 (7.2)	512 (7.1)	0.974
Chronic renal disease = Yes (%)	201 (2.8)	185 (2.6)	0.439

**Table 2:** Rates of acute myocardial infarction, hazard ratio of tooth scaling among 50–64-year-old patients vs. control patients without tooth scaling and those participants expenditure.

	Tooth Scaling Group	Comparison Group
n	7164	7164
Acute myocardial infarction = Yes (%)	136 ( 1.9)	250 (3.5)
Hazard Ratio(95%CI)	0.543 (0.441, 0.670) ^1^	1.000 (reference)
All tooth scaling expenditure	2,916,823	0
Total exp. in dental (exclude tooth scaling)	10,098,446	1,273,043
All acute myocardial infarction (AMI) expenditure	32,419,393	58,543,492
All acute myocardial infarction (AMI) expenditure/per person share	4525.32	8171.90
Ratio of AMI expenditure/All of expenditure	1.25%	1.55%
Sum of expenditure Total expenditure	45,434,66 23,625,412,192	59,816,535 3,851,848,824

^1^*p* < 0.05.

**Table 3:** Baseline characteristics of participants in the tooth scaling subgroup.

Avg. Number Per Year	Group A	Group B	
	<1 Tooth Scaling	1–2 Tooth Scaling	*p*-Value
n	5453	1711	
Gender = male (%)	2532 (46.4)	775 (45.3)	0.426
Age (mean (SD))	56.21 (4.35)	55.76 (4.30)	<0.001
Hypertension = Yes (%)	1227 (22.5)	318 (18.6)	0.001
Diabetes mellitus = Yes (%)	579 (10.6)	162 (9.5)	0.188
Hyperlipidemia = Yes (%)	550 (10.1)	201 (11.7)	0.056
Coronary artery disease = Yes (%)	402 (7.4)	112 (6.5)	0.271
Chronic renal disease = Yes (%)	154 (2.8)	47 (2.7)	0.932
Charlson comorbidity index (%)			0.345
0	4353 (79.8)	1364 (79.7)	
1~2	840 (15.4)	278 (16.2)	
3+	260 (4.8)	69 (4.0)	

**Table 4:** Hazard ratio for acute myocardial infarction among study patients by tooth scaling subgroup and those participants’ expenditure.

	Group A	Group B	*p*-Value
Avg. number per year	<1 tooth scaling	1–2 tooth scaling	
N	5453	1711	
Acute myocardial infarction = Yes (%)	108 (2.0)	28 (1.6)	0.419
Adjusted Hazard Ratio(95%CI)	0.557(0.444, 0.698)	0.497(0.336, 0.734)	<0.001
average exp. in dental (mean (SD))	1405.05 (1627.22)	3128.87 (1441.73)	<0.001
number of visits to the dentist (mean (SD))	1.22 (0.86)	2.84 (1.16)	<0.001
